# Guideline for Urine Culture and Biochemical Identification of Bacterial Urinary Pathogens in Low-Resource Settings

**DOI:** 10.3390/diagnostics10100832

**Published:** 2020-10-16

**Authors:** Nabil Karah, Rayane Rafei, Wael Elamin, Anan Ghazy, Aula Abbara, Monzer Hamze, Bernt Eric Uhlin

**Affiliations:** 1 Department of Molecular Biology and Umeå Centre for Microbial Research, Umeå University, 901 87 Umeå, Sweden; bernt.eric.uhlin@umu.se; 2 Laboratoire Microbiologie Santé et Environnement (LMSE), Doctoral School of Science and Technology, Faculty of Public Health, Lebanese University, Tripoli 1300, Lebanon; rayanerafei@hotmail.com (R.R.); mhamze@monzerhamze.com (M.H.); 3 Broomfield Hospital, Chelmsford CM1 7ET, UK; wael.elamin@yahoo.com; 4 Department of Infection, Imperial College Healthcare NHS Trust, London W12 0HS, UK; anan.ghazy@nhs.net (A.G.); a.abbara15@imperial.ac.uk (A.A.)

**Keywords:** urinary tract infection, urinalysis, Gram-negative rods, *Escherichia coli*

## Abstract

Medical diagnosis in low-resource settings is confronted by the lack of suitable guidelines, protocols and checklists. Online-accessible procedural documents are difficult to find, might be mistranslated or interpreted and usually do not address the needs of developing countries. Urinalysis, one of the most frequently performed diagnostic examinations worldwide, involves a series of tests aiming to detect particular disorders, such as urinary tract infections, kidney disease and diabetes. In this guideline, we present an alternative approach for clinical laboratories with limited resources to identify common bacterial uropathogens. We propose dividing the identification plan into two levels. The implicated pathogen will first be assigned into a bacterial group, basic identification, against which a suitable panel of antimicrobial agents shall be selected for the antimicrobial susceptibility testing (AST). Characterization of the pathogen to the genus or species level, advanced identification, will then be performed to ensure correct reading of the AST results and determine the epidemiology of clinically significant pathogens. Most of the proposed steps in our guideline are tailored to meet the needs of clinical laboratories in low-resource settings. Such guidelines are needed to strengthen the capacity of regional pathology laboratories and to enhance international initiatives on antimicrobial resistance and health equity.

## 1. Introduction

Lack of practice guidelines, standard operating procedures, protocols, check lists, and flow diagrams is a major challenge faced by clinical laboratories in countries with limited resources [1,2]. Even where protocols and standards are shared online by specialized international bodies, they are usually not developed to meet the specific needs of low resource settings and are often unavailable in all required languages [3]. Implementation of internet-downloaded guidelines is often delayed or mishandled by the local staff, mainly because of complicated algorithms, unclear terminology, unavailable material, or outdated knowledge of the documents’ users [2,3]. Guidelines for laboratory medicine routines shall accommodate up-to-date information and shall present sufficient procedural details without being difficult to read or understand [4]. Local staff, both technicians and specialists, shall be engaged in writing and revising the guidelines. Authors shall use a culturally-accepted language and shall exhibit a creative ability to include setting-tailored methodology. Although compliance with the guidelines is commonly required, local lab personnel shall be encouraged to identify and adapt to unorthodox solutions in line with the local markets and constrained resources.

In this article, we present a step-by-step guideline on how to perform semi-quantitative urine culture followed by two levels of biochemical testing for appropriate phenotypic identification of the most common bacterial urinary pathogens. Our guideline, covering part of the urinalysis test, is custom-made to meet the need of clinical laboratory staff in countries with limited resources. All tests should be calibrated prior to use. Performance characteristics should regularly be determined. All work must be carried out in a designated biosafety laboratory class 2 and all waste must be discarded according to standard local guidelines.

## 2. Urine Culture Using the Calibrated Loop/Surface Streak Method

Urine culture is the gold standard for diagnosing UTI. Different versions of the calibrated loop/surface streak method have been used since the 1960s to semi-quantify, isolate and start a presumptive identification of the microorganism(s) present in a urine specimen [5]. It is important to iterate that all samples should first be subject to a dipstick testing and/or microscopic examination to look for the presence of nitrites, white blood cells, red blood cells or bacteria [6,7].

### 2.1. Step-by-Step Procedure of the Calibrated Loop/Surface Streak Method

**Figure 1:**
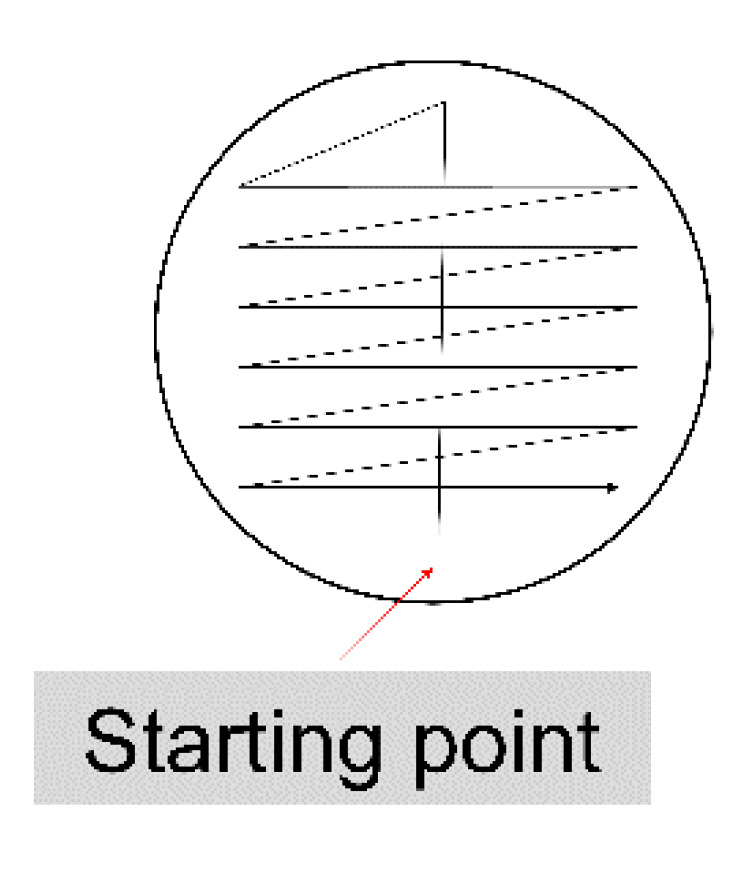
Urine culture using the calibrated loop/surface streak method.

1. Tip over the container to re-mix the urine sample.
2. Remove the cap and dip the end of a sterile 1-μL inoculating loop (white) into the urine and remove it vertically making sure that there is no urine up the loop.
3. Tip and spread the inoculum over the surface of a standard nutrient agar plate (60 × 15 mm) prepared according to the instructions of the manufacturing company.
  - Make a single streak across the centre. Then, spread the inoculum evenly distributed in a cross-zigzag arrangement to the primary streak, as shown in Figure 1.
4. Re-dip the end of the same 1-μL loop into the urine and remove it vertically making sure that there is no urine up the loop.
5. Tip and spread the inoculum over the surface of a glucose-topped MacConkey agar plate (60 × 15 mm). Spread as described above. Prepare the glucose-topped MacConkey agar plates as following:
  - Disinfect the port of a bag of 5% glucose intravenous infusion solution (1000 mL) with 70% isopropyl-alcohol-impregnated cotton ball or pad and allow to dry.
  - Aspirate 2 mL of the 5% glucose solution using a sterile needle and syringe.
  - Drop the aspirated solution on the surface of a standard MacConkey agar plate (60 × 15 mm) prepared according to the instructions of the manufacturing company.
  - Spread it by tilting the plate in different directions.
  - Leave the plate on the bench at room temperature for at least 1 h in order to allow the solution to infuse and the surface to dry.
6. Re-dip the end of the same 1-μL loop into the urine and remove it vertically making sure that there is no urine up the loop.
7. Tip and spread the inoculum over the surface of a standard MacConkey agar plate (60 × 15 mm) prepared according to the instructions of the manufacturing company. Spread as described above.
8. Incubate the plates aerobically at 35–37 °C for at 18–24 h.
9. In the following day, count the number of colonies on the surface of each medium. Each colony growing on the agar plate represents one colony forming unit (cfu)/μL (according to the size of the loop), which is equal to 1000 cfu/mL. Remember that nutrient agar is the primary medium used for counting colonies.

### 2.2. General Purpose Media Are Sufficient for Urine Culture in Low-Resource Settings

According to traditional guidelines, blood agar (non-selective medium) and MacConkey agar (selective and differential for Gram-negative rods) are probably the most commonly recommended and used media for routine urine cultures [8]. As an alternative, cysteine lactose electrolyte-deficient (CLED) agar or chromogenic agar have been proposed as standard media for urine culture [6]. Sabouraud agar should be added, in addition to the usual bacterial media, to culture the urine of patients in particular care units or if yeasts have been seen by microscopic examination [6]. In this guideline, we propose using nutrient agar, MacConkey agar, and glucose-topped MacConkey agar for the routine urine culture. However, the choice of media for routine urine culture should be made locally based on available resources and the desired approach of identification. Blood agar is replaced by nutrient agar in order to keep the costs low and since Gram-negatives have frequently accounted for the majority of anticipated pathogens.

Only if needed, blood agar will be applied as part of the Level-2 advanced bacterial identification for Gram-positives. MacConkey agar is a commonly used medium for other bacterial cultures, such as pus and cerebrospinal fluid. MacConkey agar with glucose facilitates rapid differentiation between glucose-fermenters (mainly *Enterobacterales*), which will grow as pink colonies regardless of their ability or disability to ferment lactose, and the non-fermenters (such as *Pseudomonas* spp. and *Acinetobacter* spp.), which will always grow as colourless colonies. Importantly, our protocol does not aim to undervalue or discourage the use of CLED and Chromogenic Agar. It rather provides an alternative approach.

### 2.3. Interpretation of Anticipated Results

In freshly voided urine, the culture of ≥ 100 colonies of one type (number of bacteria is ≥ 10^5^ cfu/mL) has usually been regarded as a cutoff for UTI (Table 1) [9,10]. If 10–100 colonies of one type are counted (number of bacteria is between 10^4^ and 10^5^ cfu/mL), the result should be evaluated according to the clinical status. On the other hand, the probability of UTI is low if the number of colonies is <10 (number of bacteria is < 10^4^ cfu/mL). For cultures containing two types of colonies, UTI is likely if ≥100 colonies are counted for at least one of the two types. Sub-cultures, for further identification and antimicrobial susceptibility testing, should be performed for each type counting ≥ 100 colonies, but it is also recommended to request a new sample. If both types have <100 colony, for each one, UTI is not likely and the sample is often contaminated. If there are more than two types of colonies, the sample is often contaminated. A new sample should be requested.

Nonetheless, the above-mentioned cutoffs should always be taken with caution. For instance, bacterial numbers of 10^3^ cfu/mL might be significant, especially in men, depending on the implicated pathogen [11]. Primary uropathogens, even in low amounts, could be more significant than less pathogenic bacteria in higher amounts [12]. Importantly, the occurrence of ≥10^2^ cfu/mL is an accepted criterion for UTI if the urine specimen is collected by in-and-out catheterization [13]. The use of sterile 10-μL loops (blue) is recommended in such cases in order to increase the resolution and allow quantification of lower concentrations of bacteria. When 10-μL loops are used, each colony on the agar plate equals 100 cfu/mL. It is important to emphasize that culture results should always be interpreted in line with the patient’s history, clinical symptoms and signs, and other lab findings. For instance, the quantity of squamous epithelial cells is a useful indicator of the degree of contamination from the perineal region [6].

## 3. Biochemical Identification of Common Bacterial Urinary Pathogens

Positive urine culture is usually followed by a variety of biochemical identification tests to determine the species/genus of the implicated bacterium (see Supplementary Figure S1). In this manuscript, we propose dividing the identification plan into two levels, “Basic (level 1)” and “advanced (level 2)”. Results of the basic tests shall be available one day after receiving the sample. At this level, the laboratory staff shall be able to assign the unknown pathogen into a bacterial group (*Enterobacterales*, *Pseudomonas*-like glucose-non-fermenter Gram-negative rods, *Acinetobacter*-like glucose-non-fermenter Gram-negative rods, staphylococci, streptococci, or enterococci) (Table 2). Such an assignment is needed and should be sufficient to select a suitable panel of antimicrobial agents for the AST. Results of the advanced tests shall be ready in two days after receiving the sample. These tests aim to confirm and expand the basic identification results (Table 3 and Supplementary Table S1). The advanced level will also guide a correct read of the AST results, and provide data on the epidemiology of local uropathogens.

### 3.1. Procedure of Basic Identification

1. Examine and register the ability to grow on nutrient agar and MacConkey agar plates.
2. Examine and register the ability to ferment glucose or lactose on the glucose-topped MacConkey agar plate.
3. Examine and register the ability to ferment lactose on the standard MacConkey agar plate.
4. Perform and examine a Gram-stained smear from an isolated colony.
5. For Gram-negative rods, perform and register the results of a standard oxidase test [14].
6. For Gram-positive cocci, perform and register the results of a standard catalase test [15].
7. For catalase-negative Gram-positive cocci, perform and register the results of standard pyrrolidone arylamidase (PYR) and Lancefield grouping tests according to the instructions of the manufacturing companies. Use the Lancefield grouping test mainly to detect streptococci groups B or D.

### 3.2. Procedure of Advanced Identification

#### 3.2.1. Enterobacterales

1. Perform and register the results of standard tests for indole, citrate, Voges-Proskauer, urease, hydrogen sulfide (H2S) production, motility and lysine decarboxylase (LDC) [16,17,18,19,20,21,22].
2. Retrieve the presence/absence of nitrites from the urine dipstick test.
  - The presence of nitrites indicates a positive nitrate reduction test [23].
3. If available and only when needed, perform and register the results of a commercial biochemical identification strip for *Enterobacterales*. Follow the instructions of the manufacturing company.

#### 3.2.2. Glucose Non-Fermenting Gram-Negative Rods

1. If oxidase positive, sub-culture on Cetrimide agar [24,25]. Incubate in air conditions at 35–37 °C for 18–24 h. Examine the production of pigments.
2. Perform and register the results of standard tests for indole, citrate, urease, H2S production and motility [16,17,19,20,21].
3. Retrieve the presence/absence of nitrites from the urine dipstick test.
  - The presence of nitirtes indicates a positive nitrate reduction test. However, the absence of nitrites could be due to a negative nitrate reduction test (as for *Acinetobacter baumannii*) or because nitrites have been further reduced to nitric oxide, nitrous oxide and/or nitrogen (as for *Pseudomonas aeruginosa*). If available and only when needed, perform and register the results of a standard nitrate reduction test [23].
4. If available and only when needed, perform and register the results of a commercial biochemical identification strip for non-*Enterobacterales* Gram-negative rods. Follow the instructions of the manufacturing company.

#### 3.2.3. Staphylococci (Catalase-Positive)

1. Sub-culture on blood agar and mannitol salt agar [26,27]. Place a novobiocin antibiotic disc in the centre of the first-streaked area on the blood agar plate (where you expect to see the heaviest growth). Incubate the blood agar plates in 5–10% CO_2_ and the mannitol salt agar plates in air conditions; at 35–37 °C for 18–24 h.
2. Examine and register the occurrence of complete, partial, or no hemolysis.
3. Examine and register the ability to ferment mannitol.
4. Examine and register the sensitivity to Novobiocin [28].
5. Perform and register the results of a standard rapid slide agglutination test for *Staphylococcus aureus*. Follow the instructions of the manufacturing company. It is recommended to use kits that allow a simultaneous detection of the clumping factor, protein A, and capsular polysaccharides specific for *S. aureus*.
6. For staphylococci with negative slide agglutination, perform and register the results of a standard tube coagulase test [29].
7. If available and only when needed, perform and register the results of a commercial biochemical identification strip for staphylococci. Follow the instructions of the manufacturing company.

#### 3.2.4. Enterococci (Catalase-Negative)

1. Sub-culture on blood agar and bile esculin agar [26,30]. Incubate the blood agar plates in 5–10% CO_2_ and the bile esculin agar plates in air conditions; at 35–37 °C for 18–24 h.
2. Examine and register the occurrence of complete, partial, or no haemolysis.
3. Examine and register the ability to grow on a bile medium.
4. Examine and register the ability to ferment esculin.
5. Perform and register the results of a standard 6.5% sodium chloride tolerance test [31].
6. If available and only when needed, perform and register the results of a commercial biochemical identification strip for streptococci. Follow the instructions of the manufacturing company.

#### 3.2.5. Streptococci (Catalase-Negative)

1. Sub-culture on blood agar [26]. Incubate in 5–10% CO_2_ at 35–37 °C for 18–24 h.
2. Examine and register the occurrence of complete, partial, or no haemolysis.
3. For α-haemolytic streptococci, though it is unexpected, perform and register the results of a standard bile solubility test [32].
4. If available and only when needed, perform and register the results of a commercial biochemical identification strip for streptococci. Follow the instructions of the manufacturing company.

## 4. Identification of Bacterial Urinary Pathogens in Labs with Limited Resources: Arguments for or against Particular Assays

The ability to ferment lactose, using a standard MacConkey agar medium, is included in the basic tests since bacteria from the *Enterobacterales* family are responsible for more than 50% of the cases [10]. However, lactose fermentation can be moved from “basic” into “advanced” biochemical identification. In this case, an isolated colony on nutrient agar will be sub-cultured on a standard MacConkey agar on day 1 or it can be tested using a standard carbohydrate fermentation test. The oxidase test is mainly to distinguish *Pseudomonas* spp. form the vast majority of the *Enterobacterales* bacteria as well as a number of glucose-non-fermenter Gram-negative rods (such as *Acinetobacter* spp.).

The catalase and PYR tests are included among the level-1 tests to facilitate differentiation between catalase-positive staphylococci, catalase-negative PYR-positive streptococci (mainly enterococci), and catalase-negative PYR-negative streptococci. Lancefield grouping for groups B and D tests can be used with, as a confirming test, or instead of the PYR test. The *S. aureus* slide agglutination test is included among the advanced tests although it is a rapid test and can optionally be moved from “advanced’ to “basic” biochemical identification. Our guideline states that the presence/absence of nitrites can be retrieved from the urine dipstick test. The argument behind not performing the standard nitrate reduction test, as a routine test, is related to the low added value of the second step of this test for the identification of common uropathogens. The Methyl Red test has also been excluded from our set of identification tests because it needs two days to be read the results [18], which adds an extra day to the turnaround time.

The guideline is currently under trial by two local medical laboratories in northern Syria as part of an innovative approach aiming to establish a telemedicine-supported clinical microbiology module in hard-to-reach crisis-affected regions. The hands-on time needed to perform urine culture and basic bacterial identification tests, excluding the preparation of culture media, was less than 10 min (unpublished data). The occurrence of frequent contaminations was described as a commonly faced challenge during the routine of preparing culture media. The proposed tests sufficiently and clearly differentiated between the reference strains of *Escherichia coli* (ATCC 25922), *Klebsiella pneumoniae* (ATCC 13883), *Enterobacter cloacae* (ATCC 23355), *Proteus mirabilis* (ATCC 25933), *P. aeruginosa* (ATCC 27853), *S. aureus* (ATCC 29213), and *Enterococcus faecalis* (ATCC 29212). Detailed validation of the guideline performance on clinical isolates is in progress.

## 5. Conclusions

The proposed guideline provides a tool for local medical facilities in low-resource settings to reach accurate diagnosis and deliver lab-guided treatment for patients with urinary tract infection. Such guidelines are needed to improve the quality of diagnostic services provided in countries with limited resources. The primary impact of promoting quality clinical microbiology is to reduce the mortality rates from infectious diseases. Another impact of these guidelines is to endorse application of the World Health Organisation initiatives on health equity and universal health coverage and to support local and global action plans to tackle antimicrobial resistance.

## Figures and Tables

**Table 1:** Urine culture using the calibrated loop/surface streak method. Interpretation of the urine culture results based on the morphology and number of bacterial colonies.

Morphology	Number of Colonies ^1^	Colony Forming Unit (cfu)/mL	Reading ^2^
One type	<10	<10^4^	Insignificant growth
	10–99	10^4^–10^5^	Moderate growth
	≥100	≥10^5^	Significant growth ^3^
Two types	Both < 100	Both < 10^5^	Mixed growth of two types ^4^
	One type ≥ 100	One type ≥ 10^5^	Mixed growth of two types, one is significant ^3,4^
	Both ≥ 100	Both ≥ 10^5^	Mixed growth of two types, both are significant ^3,4^
> two types	Not important	Not important	Mixed growth of several types ^4^

^1^ Culturing 1 μL urine. ^2^ Cultures without growth shall be reported as “negative” or “no growth observed”. ^3^ Sub-culture and perform antimicrobial susceptibility testing on every significant growth. ^4^ Request a new sample.

**Table 2:** Basic biochemical identification of common uropathogens.

Bacterium	Mac ^1^	Gram Stained Bacterial Cell Morphology	Glu ^1^	Oxi ^1^	Cat ^1^	PYR ^1^	Lanc ^1^
*Enterobacterales*	+	Red or pink rod-shaped	+	–	NA	NA	NA
*Pseudomonas*-like glucose-non-fermenter Gram-negative rods	+	Red or pink rod-shaped	–	+	NA	NA	NA
*Acinetobacter*-like glucose-non-fermenter Gram-negative rods	+	Red or pink rod-shaped	–	–	NA	NA	NA
Staphylococci	–	Clusters of purple or mauve sphere-shaped	NA	NA	+	NA	NA
Enterococci	–	Pairs or short chains of purple or mauve sphere-shaped	NA	NA	–	+	D
Streptococci	–	Chains of purple or mauve sphere-shaped	NA	NA	–	– *	B or D

^1^ Mac, MacConkey Agar; Glu, glucose fermentation; Oxi, oxidase; Cat, catalase; PYR, Pyrrolidone arylamidase; Lanc, Lancefield grouping; NA, Not Applicable. * Except for streptococci group A, which is not a common uropathogen.

**Table 3:** Advanced biochemical identification of common uropathogens.

*Enterobacterales* (see also Supplementary Table S1)		Lac ^1^	Ind ^1^	Cit ^1^	VP ^1^	Ure ^1^	Mot ^1^	H2S ^1^	LDC ^1^	Nit ^1^
***Escherichia coli***		+	+	–	–	–	+	–	+	+
*Klebsiella pneumoniae*		+	–	+	+	+	–	–	+	+
*Klebsiella oxytoca*		+	+	+	+	+	–	–	+	+
*Enterobacter cloacae*		+	–	+	+	V	+	–	–	+
*Enterobacter aerogenes*		+	–	+	+	–	+	–	+	+
*Citrobacter freundii*		V	–	+	–	V	+	(+)	–	+
*Citrobacter koseri*		V	+	+	–	V	+	–	–	+
*Proteus mirabilis*		–	–	V	V	+	+	+	–	+
*Proteus vulgaris*		–	+	(–)	–	+	+	+	–	+
*Providencia stuartii*		–	+	+	–	V	(+)	–	–	+
*Morganella morganii*		–	+	–	–	+	+	–	–	+
*Serratia marcescens*		–	–	+	+	(–)	+	–	+	+
**Glucose-non-** **fermenting Gram-negative rods**	**Oxi ^1^**	**Lac ^1^**	**Ind ^1^**	**Cit ^1^**	**VP ^1^**	**Ure ^1^**	**Mot ^1^**	**H2S ^1^**	**LDC ^1^**	**Nit ^1^**
*Pseudomonas aeruginosa*	+	-	-	V	-	(-)	+	-	-	V
*Acinetobacter baumannii*	-	-	-	+	-	-	-	-	-	-
**Staphylococci (catalase-positive)**		**Slide Agg ^1^**		**Tube Coag ^1^**		**Hemolysis**		**Salt Tol ^1^**	**Mann ^1^**	**Nov ^1^**
*Staphylococcus aureus*		+		+		V		+	+	S
*Staphylococcus saprophyticus*		-		-		None *		+	+ * or -	R
*Staphylococcus epidermidis* group		-		-		None *		+	-	S
**Streptococci (catalase-negative)**		**Lanc ^1^**	**Hemolysis**			**Bile esc ^1^**			**6.5% NaCl tol ^1^**	
Enterococci		D	No hemolysis *			+			+	
Group D streptococci other than enterococci		D	β-, α-, or no hemolysis			+			-	
*Streptococcus agalactiae*		B	β-hemolysis *			-			-	

^1^ Lac, lactose fermentation; Ind, indole; Cit, citrate utilization; VP, Voges-Proskauer; Ure, urease; Mot, motility; H2S, hydrogen sulfide production; LDC, lysine decarboxylase; Nit, nitrates reduction; Oxi, oxidase; Agg, agglutination; Coag, coagulase; Tol, tolerance; Mann, mannitol fermentation; Nov, novobiocin susceptibility; Bile esc, bile esculin hydrolysis; +, 90–100% Positive; -, 0–10% positive; (+), 76–89% positive; (-), 11–25% positive; V, variable.* Most strains.

## Supplementary Materials

The following are available online at https://www.mdpi.com/2075-4418/10/10/832/s1; **Figure S1.** Urinalysis chart; **Table S1.** Advanced biochemical identification of *Enterobacterales* (full list)
