# Supplementary figures and images for: Guideline for Urine Culture and Biochemical Identification of Bacterial Urinary Pathogens in Low-Resource Settings
Source: Diagnostics (Basel). 2020 Oct 16;10(10):832. doi: 10.3390/diagnostics10100832 (PMC7602787; doi:10.3390/diagnostics10100832)

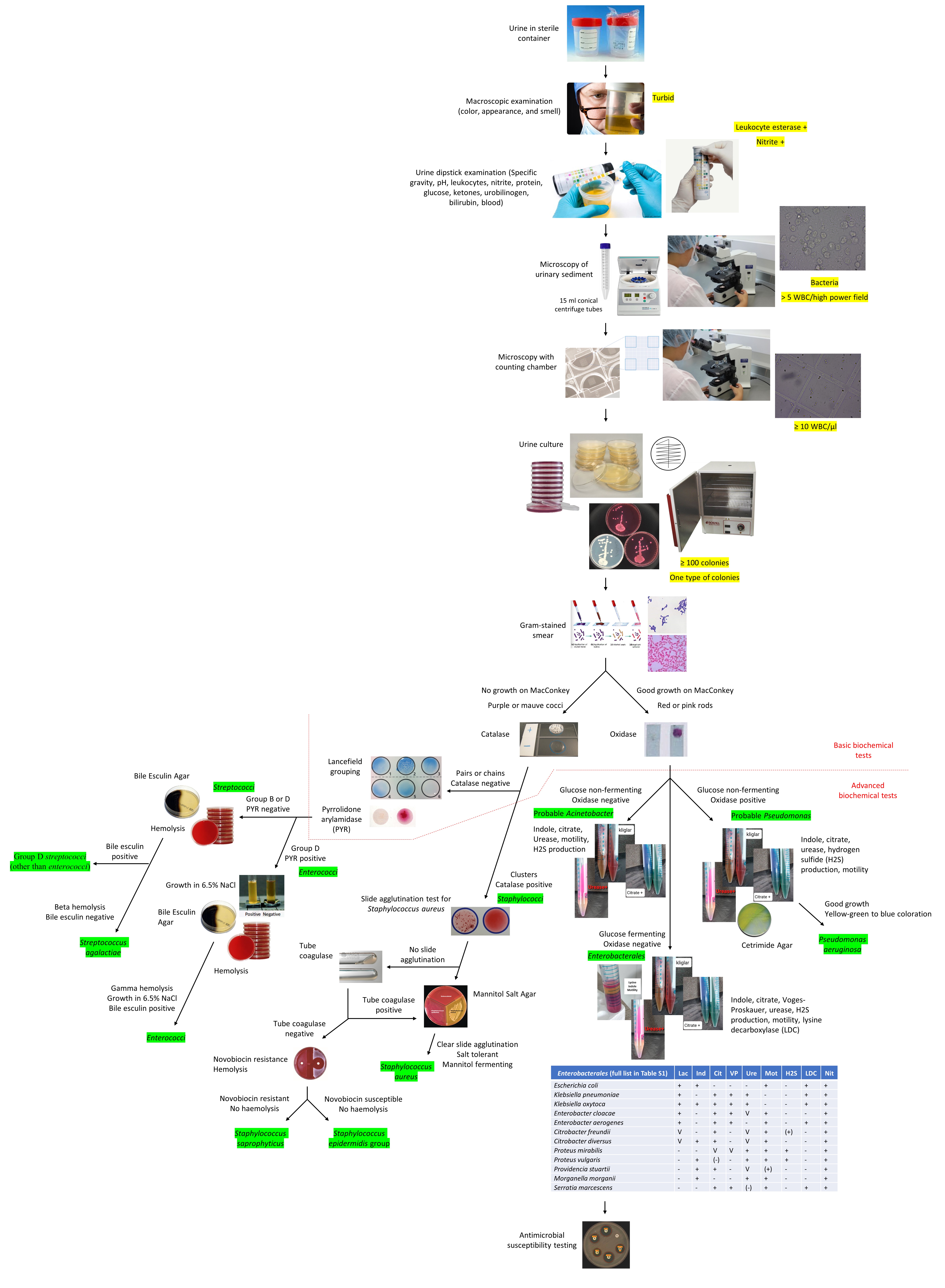

Supplement: Supplementary file 1 [file diagnostics-10-00832-s001.zip › diagnostics-925048-supplementary/20200821 Urinalysis in low-resource settings - Figure S1.jpg]
